# The biological characteristics of transcription factors AP-2α and AP-2γ and their importance in various types of cancers

**DOI:** 10.1042/BSR20181928

**Published:** 2019-03-15

**Authors:** Damian Kołat, Żaneta Kałuzińska, Andrzej K. Bednarek, Elżbieta Płuciennik

**Affiliations:** 1 Faculty of Biomedical Sciences and Postgraduate Education, Medical University of Lodz, Lodz, Poland; 2 Department of Molecular Carcinogenesis, Medical University of Lodz, Lodz, Poland

**Keywords:** cancer, TFAP2A, TFAP2C, transcription factors

## Abstract

The Activator Protein 2 (AP-2) transcription factor (TF) family is vital for the regulation of gene expression during early development as well as carcinogenesis process. The review focusses on the AP-2α and AP-2γ proteins and their dualistic regulation of gene expression in the process of carcinogenesis. Both AP-2α and AP-2γ influence a wide range of physiological or pathological processes by regulating different pathways and interacting with diverse molecules, i.e. other proteins, long non-coding RNAs (lncRNA) or miRNAs. This review summarizes the newest information about the biology of two, AP-2α and AP-2γ, TFs in the carcinogenesis process. We emphasize that these two proteins could have either oncogenic or suppressive characteristics depending on the type of cancer tissue or their interaction with specific molecules. They have also been found to contribute to resistance and sensitivity to chemotherapy in oncological patients. A better understanding of molecular network of AP-2 factors and other molecules may clarify the atypical molecular mechanisms occurring during carcinogenesis, and may assist in the recognition of new diagnostic biomarkers.

## Introduction

**Figure 1:**
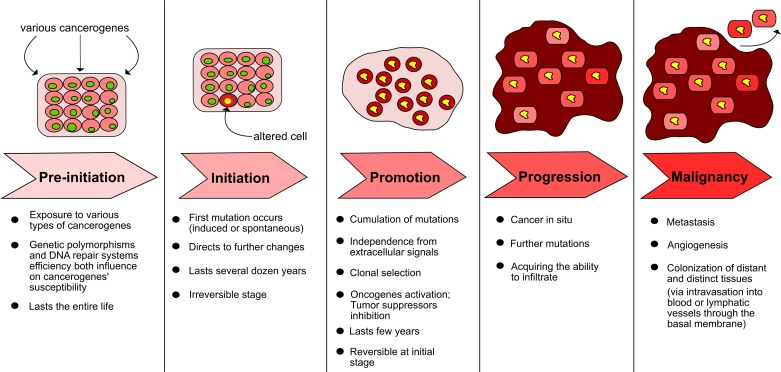
Steps of cancerogenesis (based on [3])

Genomic instability facilitates and speeds up tumor initiation and development, where several stepwise accumulations of dysfunction are implicated in the process. The acquisition of alterations; including but not limited to mutations at the nucleotide or chromosomal levels, where the appropriate repair checkpoints during cell division or at the epigenetic level are scarce, lead to multiple genetic changes. Initially the exposure of cells to tumor initiators (mutagens) and afterward tumor promoters (e.g. free radicals) remains the elementary event for tumor development [1,2]. Any deficiency of repair mechanisms or programmed cell death causes further errors present in metabolic processes or the control of cell division. Simultaneous activation of protooncogenes accompanied with suppressor gene inactivation or proliferation factor biosynthesis result in phenotypic changes in cells, followed by cancer cell infiltration and metastasis [3]. The steps of tumor development are presented in Figure 1.

In addition to the number of basic factors known to play a role in cancer development [4], several theories exist regarding the process itself, three of which are the theory of Nature and Nurture [5], the Stem Cell Theory [6], and the Mutation/Epigenetic Theory, which illustrates the potential of epigenetic processes to mediate exposure–phenotype relationships [7]. Although these hypotheses are very informative, we focussed on the one described below.

Molecular paradigm concerning suppressors and oncogenes, or more precisely Somatic Mutation Theory (SMT) [8], is the hypothesis that arises in its original form in 1914 [9]. It proposes that carcinogenesis is driven by up- or down-regulation of alterations in cancer-related genes, two key examples being tumor suppressor genes (TSGs) and oncogenes (OCGs), resulting in changes in proliferation or apoptosis [10]. However, some cancer regulatory genes display both oncogenic and suppressive traits: for example, transcription factors (TFs) [11] such as the p53 protein that can function properly only if there are no mutations in any of its subunits or any Dominant-Negative Effect (DNE) [12]. Another example is Activator Protein 2γ (AP-2γ), a member of AP-2 family of TFs, that functions as an oncogene if localized in the nucleus, but this can be inhibited by interaction with WW Domain Containing Oxidoreductase (WWOX) suppressor [13]. On the other hand, the functioning of AP-2γ it has been found to act as an inducer of p21 protein expression, suggesting that it may suppress tumor development [14]. Similarly, the AP-2α protein also possesses duality of action depending on the affected signaling pathway [15,16]. The present review examines the effect of the biological functions of two members AP-2 TFs family: AP-2γ and AP-2α on cancers development and any accompanying phenomena.

## General information about TFs

TFs can be considered in terms of mechanistic, functional, or structural properties. Structural classification of TFs is based on concurrent information about tertiary structure and sequence resemblance [17]. Briefly, there are ten distinguished sequence-homological superclasses of TFs, with 90% of human TFs fitting within the first three [18]. Further subdivisions, together with chosen examples, are given in Table 1.

**Table 1:** Superclasses of TFs (based on [19] and websites: http://gene-regulation.com/pub/databases/transfac/cl.html and http://tfclass.bioinf.med.uni-goettingen.de/)

Superclass number	Superclass name	Quantity of classes	Quantity of families	Examples of TFs
0	Yet undefined DNA-binding domains (Superclass ‘0’)	5	10	PSPC1, RFXANK, NRF1
1	Basic Domains	3	18	c-Jun, c-Fos, Nrf2
2	Zinc-coordinating DNA-binding domains	8	25	ER, GATA-1, RXR-α
3	Helix–turn–helix	7	22	HOXA9, Oct-3/4, E2F-1
4	Other all-α-helical DNA-binding domains	2	8	SOX2, TCF-7, UBF
5	α-Helices exposed by β-structures	2	7	MEF2, SRF, AIRE
6	Immunoglobulin fold	7	16	RelA, STAT1, p53
7	β-Hairpin exposed by an α/β-scaffold	2	3	SMAD4, GCM1, NF-1A
8	β-Sheet binding to DNA	2	2	TBP, TBPL1, HMGA1
9	β-Barrel DNA-binding domains	1	1	DbpA, YB-1, YBX2

Of the basic domains, Superclass 1, is termed as basic Helix–Span–Helix (bHSH) which comprises all TF AP-2 (TFAP2) representatives: α, β, γ, δ, and ε [20]. All factors in this family are crucial for gene expression in early development, regulating processes such as apoptosis or cell cycle [21]. The N-terminal region of TFAP2 factors consists of a transactivation domain, while the C-terminus contains ∼200 amino acids and is responsible for DNA binding and dimerization [22]. The dimerization domain is located inside the DNA-binding site [23], and comprise Proline/Glutamine-rich domains along with basic α-helix and bHSH. Although bHSH is unable to bind DNA when separated from the basic domain, it is still able to dimerize two family members [24]. All members of the bHSH class can recognize specific G/C-rich sequence motifs including the evolutionarily conserved binding sites GCCN3/4GGC, GCCN3/4GGG [25] or CCCCAGGC [26]. The binding region is formed of basic leucine zipper factors (bZIP) and basic helix–loop–helix factors (bHLH), which are also observed in other classes present in the basic domain superclass [24]. The members of bHSH are very similar to those of bHLH, but display a longer loop [27].

## Activating enhancer-binding protein 2 – selected representatives

**Figure 2:**
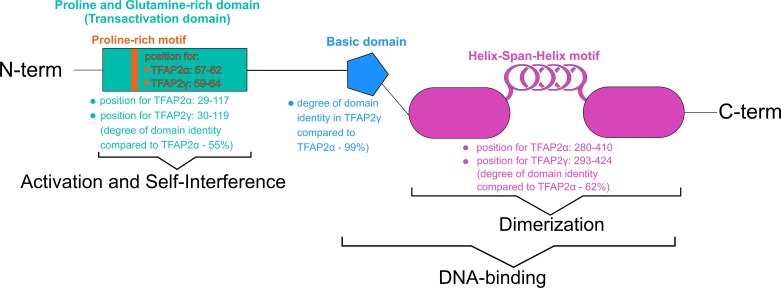
Specific domains of AP-2 family with dissimilarities amongst TFAP2α and TFAP2γ (based on [22,23,29])

The AP-2 family, within the bHSH class, is characterized by specific regions. A schematic presentation of the domains is shown in Figure 2. TFAP2δ is a peculiar case in that it displays distinct binding affinity and a lack of critical residues with a PY (proline-rich) motif as example. PPxY can interact with WWOX proteins because it is recognized by certain WW (tryptophan) domains. Moreover, modulation capabilities as negative regulation of other AP-2 factors has been suggested in the literature [28]. In terms of gene localization, the majority of mammalian family members are encoded on chromosome 6, excluding AP-2γ and AP-2ε [22]. Selected information regarding chromosomal loci and their characteristics are summarized in Table 2.

**Table 2:** Chromosomal localization and characteristics of AP-2 family members (based on information from GeneCards and NCBI ‘Gene’ databases)

AP-2 family member	Cytological location	Number of exons	Size/length	Orientation strand
*TFAP2A*	6p24.3	11	26474 bases	Minus (−)
*TFAP2B*	6p12.3	11	29910 bases	Plus (+)
*TFAP2C*	20q13.31	7	9982 bases	Plus (+)
*TFAP2D*	6p12.3	8	59490 bases	Plus (+)
*TFAP2E*	1p34.3	7	21959 bases	Plus (+)

AP-2 TFs originate in the nucleus [22]. The TFs form either homodimers or heterodimers [30]. Activity of those proteins includes modulation of transactivation potential [13], DNA-binding capability [31], degradation [32] or subcellular localization [33]; this can be achieved with the use of post-translational phosphorylation [34], sumoylation [35] or redox reactions [36]. Other proteins can either physically interact with AP-2 factors and bind to them, or only modulate their activity [22]. Furthermore, when loss of TF activity is observed due to mutation, precocious apoptosis or cell differentiation accompanied with proliferation impairment can occur [22]; however, AP-2 family members have also been found to be present at elevated levels in various types of tumors [33,37–39]. In this way, we hoped for a wider understanding of the role of TFAP2α and TFAP2γ in regulating the expression of genes that impact the clinically significant cancer phenotypes.

### AP-2α

#### Gene

The *TFAP2A* gene encodes TF which can both activate [40] and inhibit [41] transcription of other genes simultaneously. It is localized on the minus strand of chromosome 6 its heterozygous mutations, mainly deletion, but also insertion or transition, can be observed in branchio-oculofacial syndrome (BOFS) [42,43]. The region contains 26474 bases at genomic location chr6:10393186-10419659 (cytogenetic band 6p24.3; Genome Reference Consortium Human Build 38) and six mRNAs are transcribed: REFSEQ NM_001032280.2, NM_001042425.1, NM_003220.2, XM_006715175.2, XM_011514833.2, and XM_017011232.1 (NCBI Reference Sequence Database). In addition, two antisense non-coding RNA molecules have been identified (Entrez gene IDs :100130275 for TFAP2A-AS1 and 109729173 for AS2).

#### Protein

The AP-2α protein encoded by the *TFAP2A* gene may act as either a homodimer or heterodimer when working with paralogs from its family. It recognizes the specific sequence 5′-GCCNNNGGC-3′ and regulates gene transcription by interacting with enhancer elements. AP-2α is also thought to be required to preserve lens integrity after vesicle formation [44]. Four transcripts, translated into distinct isoforms, have been considered in the UniProt KnowledgeBase (identifiers: P05549-1 for canonical sequence and three variants P05549-5, P05549-2, P05549-6) and these are given in Table 3. Like most of proteins, AP-2α can undergo post-translational modification (PTM), which affect protein activity or functionality (Table 4).

**Table 3:** Comparison of AP-2α isoforms (based on UniProt KnowledgeBase)

AP-2α isoform	Length (amino acids)	Mass (Da)	Notes
Isoform ‘1’ (UniParc identifier: P05549-1)	437	48062	Canonical sequence; others refer to it
Isoform ‘2’ (UniParc identifier: P05549-5)	431	47183	Differs from canonical model in way that first 15 amino acids are substituted: MLWKLTDNIKYEDCE → MLVHSFSAM
Isoform ‘4′ (UniParc identifier: P05549-2)	365	40557	Altered amino acids from 296 to 437 as follows: EAVHLARDFG…SSDKEEKHRK → KRIHLLTRRN…SILLPSFPLP
Isoform ‘5’ (UniParc identifier: P05549-6)	433	47440	Differs from canonical model in way that first 15 amino acids are substituted: MLWKLTDNIKYEDCE → MSILAKMGDWQ

**Table 4:** Localization and effect of PTMs in AP-2α (based on GeneCards database, neXtProt platform PhosphoSitePlus resource)

Type of PTM	Position in protein	Notes (if available)
Sumoylation	10 (lysine)	Leads to inhibition of transcriptional activity
Phosphorylation	73 (tyrosine)	*None*
Phosphorylation	119 (serine)	*None*
Sumoylation	177 (lysine)	*None*
Phosphorylation	181 (serine)	*None*
Sumoylation	184 (lysine)	*None*
Phosphorylation	185 (serine)	*None*
Phosphorylation	187 (serine)	*None*
Phosphorylation	219 (serine)	Regulate molecular association and activity; induced transcription process
Phosphorylation	239 (serine)	*None*
Phosphorylation	258 (serine)	Regulate molecular association; altered transcription process
Mono-methylation	263 (arginine)	*None*
Phosphorylation	326 (serine)	*None*
Phosphorylation	333 (threonine)	*None*
Phosphorylation	428 (serine)	*None*
Phosphorylation	429 (serine)	Induced transcription process

### AP-2γ

#### Gene

*TFAP2C* is the third member of the AP-2 family and is expressed as a sequence-specific TF that activates a number of developmental genes responsible for eyes, face, and limbs formation or neural tube development. It is located on the plus strand on chromosome 20 and transcribes only one mRNA variant (NCBI Reference Sequence Database, REFSEQ accession number: NM_003222.3) and to contain 9982 bases at genomic location chr20:56629302-56639283 (cytogenetic band 20q13.31 - Genome Reference Consortium Human Build 38).

#### Protein

The AP-2γ TF shares the same properties with TFAP2α with regard to dimer formation, recognition of consensus sequence, and its effect on both cellular and viral enhancers. Only the canonical protein sequence and one isoform are given in the UniProt KnowledgeBase: Q92754-1 and Q92754-2, respectively (Table 5). Similar to AP-2α, it is modified after translation, however only one of them has a biological effect (Table 6).

**Table 5:** Comparison of AP-2γ isoforms (based on UniProt KnowledgeBase)

AP-2γ isoform	Length (amino acids)	Mass (Da)	Notes
Isoform ‘1’ (UniParc identifier: Q92754-1)	450	49,177	Canonical sequence; others refer to it
Isoform ‘2’ (UniParc identifier: Q92754-2)	281	31,010	Differs from canonical model in way that first 169 amino acids are missing

**Table 6:** Localization and effect of PTMs in AP-2γ (based on GeneCards database, neXtProt platform PhosphoSitePlus resource)

Type of modification	Position in protein	Notes (if available)
Sumoylation	10 (lysine)	Leads to inhibition of transcriptional activity
Phosphorylation	252 (serine)	*None*
Mono-methylation	276 (arginine)	*None*
Phosphorylation	434 (serine)	*None*
Phosphorylation	438 (serine)	*None*
Ubiquitylation	444 (lysine)	*None*
Ubiquitylation	447 (lysine)	*None*

## AP-2 interactions with proteins, long non-coding RNA, and miRNA molecules

### Proteins

Both the AP-2α and AP-2γ proteins play an essential role in many important biological processes. Mutations in *TFAP2A* are known to be linked to retinal defects and a greater possibility of disturbances in eye development [45]. This gene is also important in face or limb development since its expression is observed during frontal nasal process (FNP), paired lateral nasal processes, and limb bud mesenchyme (LBM) [46]. Other processes involving both *TFAP2A* and *TFAP2C* functionality concerns generation of neural tube [47,48] or body wall [49]. However, while *TFAP2C* is a gene implicated in inhibition of somatic differentiation in germ cells [50] or repression of neuroectodermal differentiation and pluripotency maintenance [51], *TFAP2A* plays a key role in kidney development [52]. The abnormal expression of *TFAP2A* may lead to BOFS and anophthalmia-microphthalmia syndrome [53], while human placenta defects are associated with *TFAP2C* overexpression [54]. The participation of AP-2α and AP-2γ in developmental processes, along with management of other events by means of specific interactions with proteins are presented in Table 7.

**Table 7:** Influence of AP-2α and AP-2γ factors on selected developmental processes, diseases, and interactive molecules (based on GeneCards, Reactome databases, and Atlas of Genetics and Cytogenetics in Oncology and Haematology)

	AP-2α	AP-2γ
Participation in development processess – major	Face, eye, limb, body wall, neural tube development	
Participation in development processess – other	Early morphogenesis of lens vesicle; kidney development	Male gonad development
Associated diseases	BOFS; ectopic thymus; anophthalmia-microphthalmia syndrome	Exencephaly; melanoma; pre-eclampsia
Mutual interactions	Interacts with WWOX, CITED2, CITED4, UBE2I, KCTD1, KCTD15, EP300; Suppresses MCAM/MUC18, C/EBPα; Stimulates transcriptional activation of PITX2	
Exclusive interactions	Along with binding NPM1 – represses HSPD1 gene expression, inhibiting formation of mitochondrial chaperonin;	Along with MTA1 – mediates epigenetic regulation of *ESR1* expression in breast cancer (BCa); Interacts with KDM5B
	Stimulates APOE gene transcription in co-operation with DEK;	
	Interacts with RALBP1 (in complex containing NUMB and EPN1) during interphase and mitosis	

Both AP-2α and AP-2γ interact with WWOX protein [44,55] while the latter is described in more detail, probably due to lower affinity of the AP-2α PPxY motif (^59^PPPY^62^) than the AP-2γ PPxY motif (^56^PPPYFPPPY^64^) [13]. The binding of AP-2 factors by WWOX suppresses their transcriptional transactivation, causing sequestration of proteins in the cytoplasm [56], thus reducing their oncogenic activity by triggering their redistribution from the nucleus [13]. It was also suggested that modulation of both α and γ AP-2 factors could be important from clinical point of view [56].

In addition to the examples summarized above, three interactions considering proliferation and cell cycle regulation are worth mentioning. First, the AP-2γ homodimer is capable of binding the region of *EGFR* gene promoter, therefore stimulating its expression and regulating tumor growth and survival in HER2 positive breast cancer patients [57,58]. However, when it comes to AP-2α, it acts conversely to AP-2γ in terms of proliferation but with a different target. *VEGFA* gene promoter consists of response elements recognized by AP-2α and, as a result, its expression is inhibited [15].

**Figure 3:**
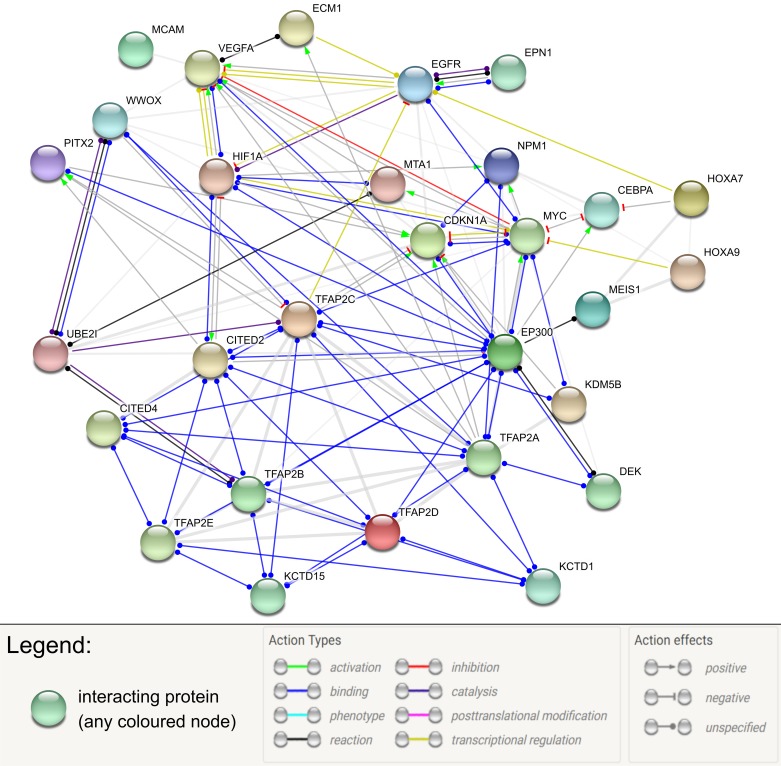
Interaction network of AP-2 factors with selected proteins (STRING database)

Finally, the cell cycle can be controlled by Cyclin-Dependent Kinase Inhibitor 1A (CDKN1A) regulation, but its inhibition or stimulation depends on the AP-2 member. TFAP2α is thought to stimulate *CDKN1A* by direct interaction with two response elements in its promoter [59,60]. In contrast, the formation of the AP2γ homodimer provides a possibility to interact with the previously mentioned KDM5B along with MYC. This complex is thought to influence the progression of the cell cycle and carcinogenesis by repressing CDKN1A expression [61]. AP-2 factors have an abundant interaction network with other molecules and are associated with several important processes (Figure 3).

### Long non-coding RNA and microRNA

Just like proteins, AP-2 interacts with RNA. In many cases, this molecular feedback regulates cancer progression, tumor growth, metastasis, drug resistance, metabolism, or even the occurrence of congenital anomalies.

Long non-coding RNA (lncRNA) derived from the growth arrest-specific 5 (*GAS5*) gene has been found to participate in progression of glioma. In the promoter region, the functional polymorphism (rs145204276) with genetic variation in the presence or absence of specific sequence (insertion-deletion, abbreviated in/del) may influence lncRNA expression by recognition by recruited TFs. The most abundant TFs were three members of the AP-2 family – *TFAP2A, B*, and *C*, with *TFAP2A* gene expression correlating positively with *GAS5* expression in glioma [62]. In a different case, progression of colorectal cancer (CRC) was found to be associated with down-regulated AP-2α expression through direct targetting by a CRC-associated lncRNA (CAAL) molecule [63]. The down-regulation of AP-2α had an impact on Wnt/β-catenin pathway which was activated in the presence of the key regulator CCAL; this phenomenon was also observed in hepatocellular carcinoma (HCC) [64]. In fact, the entire regulatory axis could be valuable in CRC-targetted therapy: lncRNA UCA1 is also up-regulated in CRC and involved in cellular migration, its expression correlates with that of both *TFAP2A* and *TFAP2C*, and its dependence relies on the occurrence of TF-binding sites (TFBSs) that are located upstream of the start site of UCA1 transcription [65].

An example of a molecule that maintains the balance between pluripotency and differentiation is lncPRESS1: an lncRNA that displays control over the expression of TFAP2C and other pluripotency genes in human embryonic stem cells (hESCs). lncRNA depletion leads to differentiation by altering pluripotency and down-regulating associated genes [66]. It has been proposed that a group of lncRNAs responsive to oxygen-glucose deprivation (OGD) may act as mediators of ischemic stimuli to the endothelium and that TFAP2C is one of the TFs affecting OGD-responsive lncRNAs, such as lnc-OGD4751, under specific conditions [67].

In the case of miRNA, most of the molecules described herein have been identified as regulators of tumor progression or treatment resistance. In melanoma cell lines, two miRNA molecules interacting with AP-2 family members were indicated as being significantly vital for cancer development: miR-214 and miR-638. The former is able to influence both *TFAP2C* [68] and *TFAP2A* [69], thus contributing to melanoma progression (A375P cell line); it was found that TFAP2γ is an important factor which permits miR-214 to guide tumor progression [68]. Additionally, the abolition of *TFAP2A* expression in melanoma was confirmed to increase malignancy [70]. The latter, miR-638, has been identified as an overexpressed molecule in metastatic melanomas in relevance to primary tumor. Promoter analysis indicated that together with *TFAP2A*, they develop regulatory feedback that possesses double-negative character [71]. Suppression of p53-mediated apoptosis and autophagy combined with down-regulation of AP-2α provides favorable conditions for metastasis. Lung cancer is also modulated by a couple of miRNAs interacting with the AP-2 family. The first, miR-1254, a negative regulator of heme oxygenase-1 (HO-1), is able to induce apoptosis and inhibit cell cycle progression in non-small cell lung carcinoma (NSCLC) by a two-sided approach. First, it directly targets 3′-UTR of *HO-1* mRNA, eliminating its function in cancer, i.e. angiogenesis stimulation or inflammatory response inhibition [72,73]. Second, it prevents TFAP2α from functioning as a transactivator of HO-1, influencing the targetting via the non-seed sequence. When present, these conditions intensify the suppressive effect on the tumor [74].

However, overexpression of *TFAP2C* in NSCLC leads to excessive cell cycle activation which promotes tumor aggressiveness: the proposed model of action consists of simultaneous induction of oncogenic miR-183 and down-regulation of suppressive miR-33a. This entails, respectively, the blocking of AKAP12-mediated inhibition of cyclin D1 and the activation of cell cycle progression by cyclin-dependent kinase 6 (Cdk6) [75]. Studies of the Slug-mediated network have found that another tumor progression in NSCLC can be also be promoted by suppressing TFAP2C. Since Slug directly binds to the promoter region of miR-137, this enhances RNA expression in lung cancer cells. In turn, miR-137 suppresses AP-2γ expression directly, which increases metastasis and tumor invasion [76]. A similar pathway, but with a different effect, could be observed in neuroblastoma: miR-200a directly targets *TFAP2C*, thus tumor growth and cell proliferation [77]. Targetting AP-2 factors could as well lead to drug resistance, as confirmed in pancreatic cancer, where gemcitabine resistance is obtained via *TFAP2C* suppression by miR-10a-5p [78], or in bladder cancer where *TFAP2A* is suppressed by miR-193a-5p [79]. In both cases, this could indicate informative new therapeutic targets to remove resistance to treatment.

**Figure 4:**
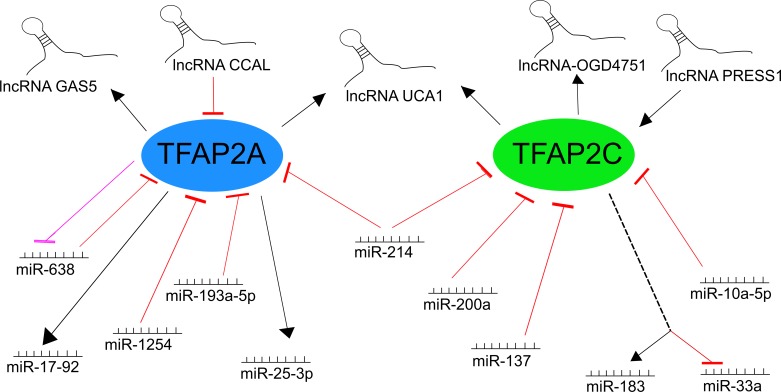
Interaction network of AP-2α/γ factors with selected lncRNA and miRNA molecules (based on [62–81])

Other miRNA-TFAP2 combinations have been observed in other contexts. AP-2α was listed as a regulator of at least two miRNAs that are responsible for either metabolism regulation or proper craniofacial phenotype (or disrupted when gene is mutated). In the first case, AP-2α binds to the core promoter of miR-25-3p, thus increasing its expression and allowing it to target the *Akt1* gene and enhance the metabolism in C2C12 cells [80]. In the second, it recognizes multiple binding sites in miR-17-92 chromatin that induce miRNA expression and allow proper midface development through regulation of *Tbx* genes. Unfortunately, occurrence of mutant miR-17-92 could result in cleft lip and palate (CL/P) which is a common congenital anomaly [81]. As clearly seen above, *TFAP2A* and *TFAP2C* are of great importance in a number of processes in which they play a controlling role or are themselves regulated by RNA molecules. A summary of the above interactions is presented in Figure 4.

## Clinical data, participation in developmental processes, and cancers

Quantitative transcriptomics analyses have found *TFAP2A* and *TFAP2C* to occur predominantly in the placenta, skin, or esophagus, and for the two to be expressed at significantly different levels in the kidney, in favor of *TFAP2A* [82]. In terms of developmental processes, abnormal AP-2α expression may result in incorrect placenta maturation in high-risk pregnancies [83]. Likewise, high expression of AP-2γ can be preventive during pre-eclampsia, via blood pressure regulation by compensation of vasoconstructory peptides excess [84]. In case of tumorigenesis, AP-2α has demonstrated protective properties against melanoma progression, which can be disrupted following cleavage by caspase-6 [85]. However, in melanoma, AP-2γ overexpression is associated with unfavorable processes including vascular invasion or increased vascularity [86]. At last, AP-2γ is up-regulated in esophageal adenocarcinoma, suggesting it may play a progressive role [87], while high AP-2α expression correlates with longer overall survival (OS) in esophageal squamous cell carcinoma and has been proposed as a valuable prognostic biomarker in this disease [88]. Apparently, those two AP-2 family members are thought to have oncogenic or suppressive characteristics depending on the cancer tissue or interaction with other molecules. The function of *TFAP2A* and *TFAP2C* in selected cancers with implicated pathways (if any) is summarized in Tables 8 and 9.

**Table 8:** Regulatory role of AP-2α in various types of cancer along with involved molecular pathways (based on [15,16,89–104])

Protein	Type of cancer	Regulation	Function	Pathways (if available)	References
**AP-2α**	Hepatocellular carcinoma	**Suppressor**	*TFAP2A* overexpression decreases cell migration and invasion, and also leads to inhibition of cell growth and proliferation	HIF-1α-mediated VEGF/PEDF signaling pathway;	[15,89–92]
				β-catenin/TCF/LEF signaling;	
				Bax/cytochrome *c*/Apaf1/caspase9-dependent mitochondrial pathway;	
				CdK-inhibitor p21^WAF^ in p53-dependent and p53-independent pathways	
**AP-2α**	Breast cancer	**Suppressor**	Reduced *TFAP2A* is associated with more aggressive breast cancer		[93,94]
			*TFAP2A* high expression is related to sensitiveness to chemotherapeutic drugs (due to massive induction of apoptosis)		
**AP-2α**	Glioblastoma	**Suppressor**	*TFAP2A* reduces tumor cell growth, increases cell death, attenuates cell migration, and endothelial tube formation		[95]
**AP-2α**	Melanoma	**Suppressor**	Comparison of stage 4 melanomas compared with non-stage 4 display, that silenced *TFAP2A* by aberrant CpG methylation of its promoter is most decreased in higher stages		[96]
**AP-2α**	Gastric cancer	**Suppressor**	*TFAP2A* can reverse the multidrug resistance (MDR)	Notch signaling pathway	[97,98]
			Lower level of *TFAP2A* leads to unfavorable prognosis for patients		
**AP-2α**	Prostate cancer	**Suppressor**	Loss of *TFAP2A* leads to decreased cellular zinc uptake which is essential for tumor development		[99]
**AP-2α**	Colorectal cancer	**Suppressor**	*TFAP2A* negatively regulates downstream targets of β-catenin/TCF/LEF	Wnt signaling pathway	[90]
**AP-2α**	Neuroblastoma	**Oncogene**	*TFAP2A* is overexpressed in cell lines derived from high-stage tumors		[100]
**AP-2α**	Pancreatic cancer	**Oncogene**	Cell lines express high nuclear levels of *TFAP2A*		[101]
			AP-2α variant 6 seems to be specific for pancreatic cancer		
**AP-2α**	Acute myeloid leukemia	**Oncogene**	*TFAP2A* affects Hoxa7, Hoxa9, and Meis1 which are involved in leukemogenesis		[102]
**AP-2α**	Squamous cell carcinomas	**Oncogene**	AP-2α is involved in complex keratinocyte biology including proliferation, differentiation, and carcinogenesis		[103,104]
**AP-2α**	Nasopharyngeal carcinoma	**Oncogene**	*TFAP2A* overexpression correlates with HIF-1α expression along with advanced tumor stage, local invasion, clinical progression, or poor prognosis	HIF-1α-mediated VEGF/PEDF signaling pathway	[16]

**Table 9:** Regulatory role of AP-2γ in various types of cancer along with involved molecular pathways (based on [38,75–77,105–108])

Protein	Type of cancer	Regulation	Function	Pathways (if available)	References
**AP-2γ**	Breast cancer	**Oncogene**	*TFAP2C* expression is associated with proliferation, disease progression, and endocrine therapy resistance		[105]
			*TFAP2C* directly represses *CDKN1A* gene thereby promoting proliferation		
			High *TFAP2C* and low *CD44* expression are associated with pathologic complete response (pCR) after neoadjuvant chemotherapy		
**AP-2γ**	Melanoma	**Oncogene**	High level of *TFAP2C* regulates *ECM1* overexpression which is associated with poor prognosis		[106]
**AP-2γ**	Testicular cancer	**Oncogene**	*TFAP2C* is a novel marker of testicular CIS and CIS-derived tumors along with its involvement in self-renewal and survival of immature germ cells and tissue-specific stem cells		[38]
**AP-2γ**	Neuroblastoma	**Oncogene**	*TFAP2C* knockdown results in inhibition of cell proliferation and tumor growth		[77]
**AP-2γ**	Primary ovarian tumors	**Oncogene**	Overexpressed in advanced-stage cancers compared with early-stage carcinomas		[107]
**AP-2γ**	Lung cancer	**Oncogene**	*TFAP2C* overexpression promotes cell viability, proliferation, and cell cycle progression	AK1 signaling MAPK and Snail pathways activated by TGFBR1-PAK1 signaling	[75,108]
**AP-2γ**	Lung cancer	**Suppressor**	*TFAP2C* blocks AKAP12-mediated cyclin D1 inhibition (by inducing the overexpression of oncogenic miR-183)		[76]
			*TFAP2C* activates Cdk6-mediated cell cycle progression (by down-regulating tumor-suppressive miR-33a)		
			Patients with low-level expression of Slug and miR-137 but high-level expression of *TFAP2C* experienced better survival		

Moreover, these TFs are essential for chemotherapy sensitivity in patients. *In vitro* study demonstrates that AP-2α increases the sensitivity to cisplatin in endometrial cancer cells, and also proving that single nucleotide polymorphism (SNP) rs1045385 A>C variation in the 3′-UTR region of the *TFAP2A* gene could become a prognostic marker in the treatment of cisplatin. This is due to the fact that only the A allele is recognized by miR-200b/200c/429 family which reduces the expression of the AP-2α suppressor thereby correlating with resistance to cisplatin [109]. Furthermore, studies based on siRNA against AP-2α in MDA-MB-231 cells have shown that the use of 5-aza-2′-deoxycytidine alone for breast cancer treatment does not increase cell apoptosis or cancer sensitivity to chemotherapy; however, proper expression of *TFAP2A* combined with chemotherapy resulted in loss of tumorigenesis, reduction in colony formation, and heightened chemosensitivity [110]. At last, AP-2α is thought to be valuable in gastric cancer during occurrence of multidrug resistance (MDR). Using a vector overexpressing AP-2α in SGC7901/VCR cells, Lian et al. [97] demonstrated inhibition of the Notch signaling pathway which could potentially play a role in reversing drug resistance. Additionally, increased cell cycle arrest (G_0_/G_1_ phase) and apoptosis were observed during AP-2α overexpression [97]. In turn, the role of *TFAP2C* in terms of resistance to treatment appears to be reversed in comparison with *TFAP2A*. In patients with invasive breast cancer, both *in vitro* and clinical experiments indicated that AP-2γ overexpression determines resistance to tamoxifen or faslodex, which is possible due to inhibition of growth arrest and thus a worse outcome [111].

## Conclusion

Both *TFAP2A* and *TFAP2C* hold distinct roles as oncogenes or tumor suppressors in various tumor models. However, the former tends to play a protective role, while the latter is predominantly up-regulated and participates in pathways responsible for cancer progression. Although AP-2α and AP-2γ are generally similar with regard to their activities, they do differ in a number of ways. Both act by regulating the specific expression of many genes which are frequently related to tumorigenesis, together with their products: proteins, lncRNAs, and miRNAs. Key examples of these products are p21, WWOX, EGFR, VEGF-A, lncRNA GAS5, lncRNA UCA1, miR-183 or miR-638. Further studies are needed to better understand the molecular network associated with AP-2 members, thus providing access to a wider range of elementary biomarkers which can be used to improve cancer diagnosis and treatment.

## Abbreviations

AP-2: activator protein 2
bHLH: basic helix–loop–helix
bHSH: basic helix–span–helix
BOFS: branchio-oculofacial syndrome
bZIP: basic leucine-zipper factor
CCAL: colorectal cancer-associated lncRNA
CDKN1A: cyclin-dependent kinase inhibitor 1A
CRC: colorectal cancer
DNE: dominant-negative effect
FNP: frontal nasal process
GAS5: growth arrest-specific 5
HCC: hepatocellular carcinoma
hESC: human embrionic stem cell
HO-1: heme oxygenase-1
LBM: limb bud mesenchyme
lncRNA: long non-coding RNA
MDR: multidrug resistance
NSCLC: non-small cell lung carcinoma
OCG: oncogene gene
OGD: oxygen-glucose deprivation
OS: overall survival
PTM: post-translational modification
SMT: somatic mutation theory
TF: transcription factor
TFAP: transcription factor activator protein
TFBS: transcription factor-binding site
TSG: tumor suppressor gene
WWOX: WW domain containing oxidoreductase
